# Color harmony represented by activity in the medial orbitofrontal cortex and amygdala

**DOI:** 10.3389/fnhum.2015.00382

**Published:** 2015-07-01

**Authors:** Takashi Ikeda, Daisuke Matsuyoshi, Nobukatsu Sawamoto, Hidenao Fukuyama, Naoyuki Osaka

**Affiliations:** ^1^Graduate School of Engineering, Osaka UniversitySuita, Japan; ^2^Graduate School of Medicine, Osaka UniversitySuita, Japan; ^3^Graduate School of Letters, Kyoto UniversityKyoto, Japan; ^4^Research Center for Advanced Science and Technology, The University of TokyoTokyo, Japan; ^5^Graduate School of Medicine, Kyoto UniversityKyoto, Japan

**Keywords:** color harmony, fMRI, neuroesthetics, orbitofrontal cortex, amygdala, insula

## Abstract

Observing paired colors with a different hue (in terms of chroma and lightness) engenders pleasantness from such harmonious combinations; however, negative reactions can emerge from disharmonious combinations. Currently, neural mechanisms underlying the esthetic and emotional aspects of color perception remain unknown. The current study reports evidence regarding the neural correlates of color harmony and disharmony. Functional magnetic resonance imaging was used to assess brain regions activated by harmonious or disharmonious color combinations in comparison to other stimuli. Results showed that the left medial orbitofrontal cortex (mOFC) and left amygdala were activated when participants observed harmonious and disharmonious stimuli, respectively. Taken together, these findings suggest that color disharmony may depend on stimulus properties and more automatic neural processes mediated by the amygdala, whereas color harmony is harder to discriminate based on color characteristics and is reflected by the esthetic value represented in the mOFC. This study has a limitation that we could not exclude the effect of preference for color combination, which has a strong positive correlation with color harmony.

## Introduction

**Figure 1:**
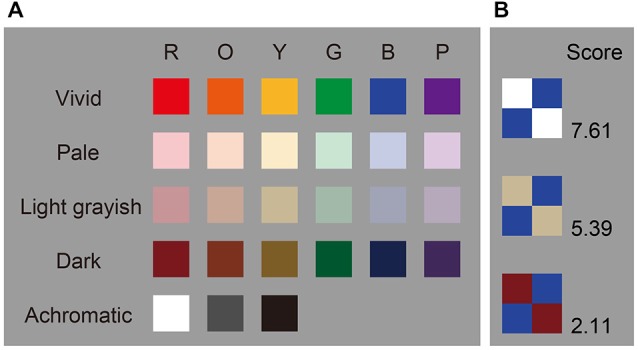
**Color pallets and examples. (A)** Color pallets used in this study. Color coordinates are shown in Table 1. **(B)** Examples of color combination stimuli. Scores indicate average color harmony ratings for the 18 participants.

It is well established that individuals tend to gravitate toward harmonious color combinations as a source of general comfort. This can be reflected in the clothing combinations we select, the interior décor we prefer, and so on. Research on color theory has a long history. For example, Theory of Colors, a book published by Goethe in 1810, established a wheel describing principal components of colors, which consist of three primary (yellow, red, and blue) and three secondary colors (orange, green, and violet); it was thought that harmonious relationships existed between colors opposite (complementary) each other on this wheel (Von Goethe, 1970). Additionally, Munsell (1907) developed a color order system that was based on three perceptual properties: hue, value (lightness), and chroma (saturation). With the development of this system, all perceptible colors could be accommodated in a slightly distorted spherical solid. Based on this system, Moon and Spencer (1944) proposed identity, similarity, and contrast as three principles for color harmony, while Judd and Wyszecki (1963) proposed four principles of color theory including order, familiarity, similarity, and unambiguity. Both principles suggest that harmony is created when neighboring colors share similarities or contrast in hue. More recent studies have focused on how to generate harmonious color combinations based on hue, saturation, and lightness. These psychological studies have sought to validate and predict harmony scores based on these three properties (Ou and Luo, 2006; Szabó et al., 2010; Ou et al., 2011). These studies used CIELAB to quantitatively assess differences in uniform color space. The CIELAB system has three perceptually even-interval scales, which include lightness (L*), red-green (a*), and blue-yellow (b*). Additionally, chroma (C*) and hue (H*) are calculated from the length and angles of vectors used on the a*b* plane, respectively. This system aims to identify uniform changes in perceived color. Figure 1 depicts a color palette with examples of harmonious, neutral, and disharmonious color combinations. A pleasing effect is strongly determined by the neighboring color.

However, the word “pleasing” might be a bit loaded. Schloss and Palmer (2011) pointed out that color harmony judgments tend not to be clearly separated from color preference judgments in previous studies. They proposed three methods for evaluating color combination stimuli: pair preference, pair harmony, and figural preference. According to traditional theories, a combination (which has a large contrast in hue) tends to be harmonious, but it relies on figural preference instead of pair harmony or pair preference. Both pair preference and harmony ratings negatively correlate with hue contrast. A pair preference rating increases when a combination has a large contrast in lightness, and component colors used in combination are preferred. Although harmony and preference are considered positive affect labels, we should treat them as different judgments.

Correlation coefficients between harmony and preference are very high (e.g., *r* = 0.80) at the group level. However, individual correlation coefficients range from −0.54 to 0.86 (Palmer and Griscom, 2013). This suggests that a tendency toward liking a harmonious combination (“preference for harmony:” Schloss and Palmer, 2011; Palmer and Griscom, 2013) is based on individual differences. Moreover, preference for harmony decreases with artistic training.

These early studies revealed that color harmony could be predicted by these three properties. However, the neural mechanisms underlying the esthetic and emotional aspects of color, specifically harmony or disharmony, are still poorly understood. There is evidence that color information is first processed in the retina followed by higher visual cortices (McKeefry and Zeki, 1997; Bartels and Zeki, 2000; Winawer et al., 2010). Since color harmony has been reported to exert pleasing effects (Judd and Wyszecki, 1963), brain regions activated by harmonious or disharmonious color combinations should be processed in terms of both perceptual and affective features, particularly in terms of their esthetic aspects. Furthermore, disharmonious color combinations might elicit an opposite, negative emotion (perhaps more in line with amygdala).

Previous studies have indicated that color harmony can be influenced by many factors such as shape, size, number of colors, and relative positions of colors in a combination (Hård and Sivik, 2001; Burchett, 2002). Therefore, the current study first examined the association between perceptual properties and color-harmony scores with 351 color pair combinations presented against a gray background. The use of different color combination pairs allowed for the assessment of relationships between two colors and their three perceptual properties: lightness, chroma, and hue. Functional magnetic resonance imaging (fMRI) was used to examine the brain regions activated during the presentation of harmonious and disharmonious combinations compared with other stimuli.

## Materials and Methods

### Participants

Eighteen healthy right-handed volunteers (6 females and 12 males, aged 19–30) participated in the preliminary psychophysical and fMRI experiment. All participants had normal or corrected-to-normal vision, and none had a history of neurological or psychiatric disorders. Moreover, none of the participants had any special experience with art or color design. We used Ishihara plates for the color vision test under a fluorescent light simulating D65 illuminant. Written informed consent was obtained from all participants, the experiments were conducted in accordance with the ethical guidelines of the Declaration of Helsinki, and all methodology was approved by the Committee of Medical Ethics, Graduate School of Medicine, Kyoto University. Both experiments were conducted in a darkened room.

### Preliminary Psychophysical Experiment

**Figure 2:**
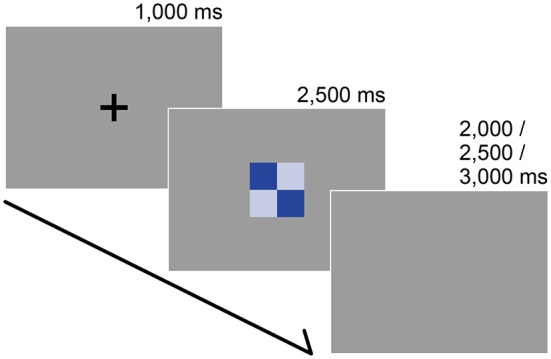
**Stimuli and trial sequence**. The participants’ task was to report the subjective color harmony score. Each trial began with a black fixation that was presented for 1000 ms. After a color combination appeared, participants were instructed to rate the combination as quickly as possible along a 9-point scale (1 = disharmony, 9 = harmony) during a preliminary psychophysical experiment or on a 3-point scale (1 = disharmony, 3 = harmony) within 2500 ms during the fMRI experiment. The inter-trial interval was randomized.

We made a color palette that contained 27 colors (six hues across four tones and three achromatic colors, see Figure 1). These colors were chosen from the Practical Color Co-ordinate System (PCCS: developed by Japan Color Research Institute), which has been determined to be suitable for making harmonious color combinations. Table 1 shows the color coordinates in the CIELAB color space measured by a luminance colorimeter (BM-5A, TOPCON, Japan) based on the LCD display (LCD2690WUXi, NEC, Japan) employed here. Finally, we made 351 color combination pairs that were arranged in 2 × 2 checkerboard pattern (Figure 2). We presented these as stimuli in the center of the display against a neutral gray background, which was controlled using Presentation software (version 12.2, Neurobehavioral Systems Inc., San Francisco, CA, USA) running on Windows XP. The distance between a participant and the display was 80 cm and the height and width of a combination stimulus was 8° in the visual angle, respectively. During the experiment, the participant was asked to rate the stimulus as soon as possible along a 9-point scale (1 = disharmony, 9 = harmony), using nine numerical keys on a keyboard. Participants’ rating criteria included “harmony of the two-color combination”; therefore, they were instructed neither to observe just one color nor to rate along “like–dislike” dimension, since preference for the pair as a whole, harmony of the pair as a whole, and preference for its figural color could yield different results (Schloss and Palmer, 2011). In total, participants rated 702 stimuli since we flipped the two colors (351 combinations × 2 configurations) to avoid influences of spatial bias. Before the actual experiment, subjects participated in 60 training trials using color combination stimuli that were not used during the study.

**Table 1:** **Color coordinates for each stimulus in the CIELAB color space**.

Color	L*	a*	b*	C_ab_*	h_ab_*
vR	47.27	58.07	30.21	65.45	27.48
vO	66.72	14.85	62.08	63.84	76.55
vY	81.52	−9.77	71.13	71.79	97.82
vG	62.51	−39.70	23.85	46.31	149.01
Vb	44.96	14.99	−56.67	58.62	255.19
vP	35.60	46.19	−32.63	56.55	215.24
pR	86.83	8.04	4.25	9.10	27.84
pO	89.08	1.38	12.70	12.77	83.80
pY	90.81	−4.62	18.56	19.13	103.96
pG	85.99	−13.15	6.54	14.69	153.56
pB	82.71	−0.30	−8.48	8.49	272.04
pP	82.58	5.91	−3.52	6.88	210.81
ltgR	70.93	8.13	2.50	8.51	17.11
ltgO	76.69	4.34	10.05	10.94	66.66
ltgY	77.56	−1.60	16.38	16.46	95.57
ltgG	71.33	−11.18	6.64	13.00	149.31
ltgB	66.27	1.15	−8.69	8.76	262.47
ltgP	66.56	6.75	−3.72	7.71	208.84
dkR	26.00	27.63	8.34	28.86	16.80
dkO	35.97	13.85	30.10	33.13	65.29
dkY	42.66	2.02	35.82	35.88	86.77
dkG	30.80	−19.24	11.62	22.48	148.88
dkB	20.50	9.20	−30.09	31.47	252.99
dkP	21.69	19.39	−15.32	24.71	218.32
White	100.00	0.00	0.00	0.00	–
Gray	40.86	1.86	−0.21	1.87	186.56
Black	11.37	−1.02	−2.18	2.41	295.09
Background	70.53	3.98	−0.21	3.98	182.99

*The white point was calculated from an emulated D65 standard illuminant on the LCD display and the screen. (v, vivid; p, pale; ltg, light grayish; dk, dark; R, red; O, orange; Y, yellow; G, green; B, blue; P, purple)*.

### fMRI Experiment

The fMRI experiment was conducted in a 3-T MRI scanner (Trio, Siemens, Germany). A forehead strap and form pads were used to reduce head motion. Functional images were obtained using a gradient-echo echo-planar pulse sequence (TR = 2500 ms, TE = 30 ms; flip angle = 90°, voxel size = 3 mm × 3 mm × 3 mm; 36 axial slices). To minimize signal loss in the orbitofrontal cortex (OFC) caused by local susceptibility gradients, we used a tilted acquisition sequence at 30° to the AC-PC line, and sufficient signal quality in orbitofrontal and amygdala regions was acquired in the test run. Following the acquisition of functional images, anatomical T1-weighted images (MPRAGE sequence, voxel size = 0.94 mm × 0.94 mm × 1 mm, 208 slices) were collected.

From the results of the preliminary psychophysical experiment, we made individual stimulus sets for each participant to optimize the subjective experience of color harmony and disharmony; we selected 30 stimuli in each condition according to the highest, middle, and lowest given scores individually. We defined two criteria: a difference between the first and second rating scores were within 2-points or a reaction time ≥2500 ms. If a combination stimulus did not satisfy these criteria, we excluded it from the individual data set and included another stimulus that met these criteria.

In the fMRI experiment, participants viewed stimuli on a screen projected (U2-X2000, PLUS Corporation, Japan) through a mirror attached to the head coil. Earplugs were used to reduce the noise from the MRI scanner. We calibrated the projector using a luminance colorimeter (CS-100A, Konica Minolta, Japan) in order to set to the same CIELAB color coordinates that were used during the preliminary psychophysical experiment. Therefore, color appearance in both the experiments remained the same.

Following the presentation of a black fixation cross (for 1000 ms), participants were instructed to rate a stimulus on a 3-point scale (1 = disharmony, 3 = harmony) within 2500 ms by pressing three buttons on the response box in their right hand. After the rating, we set a pseudo-randomized inter-trial interval (2000, 2500, or 3000 ms) before starting the next trial (Figure 2). Participants rated for 180 trials (30 stimuli × 3 data sets × 2 repetitions) and 60 catch trials in which they had to press any button as soon as the black fixation point turned white. All trials were presented in a pseudo-randomized order.

### Image Processing and Analysis

Image processing and analysis were performed using SPM8 (Wellcome Department of Cognitive Neurology, UK) running on MATLAB (MathWorks Inc., Sherborn, MA, USA). First, we conducted slice acquisition timing correction to functional images. These images were realigned to the mean image for correction of head movement. T1-weighted anatomical images were then normalized to the Montreal Neurological Institute (MNI) space, and its parameter was applied to normalize the realigned functional images. Images were then smoothed with an isotropic Gaussian kernel of 9-mm full width-half maximum, and low-frequency noise was removed using a high-pass filter (time constant 128 s).

Individual analysis was performed with a fixed effect model. Statistical parametric maps were calculated to identify voxels with event-related BOLD signal changes using the general linear model (GLM). Trials were classified into three conditions (Harmony, Neutral, and Disharmony) by the responses of each participant during functional scanning. Each event defined as the onset of a stimulus presentation was convolved with a canonical hemodynamic response function (HRF) to provide regressors for the GLM. Reaction time was entered as a first-order parametric modulator. Head movement parameters calculated in the realignment step and onsets of the catch trials convolved with HRF were included in this model as regressors of no interest. Lastly, contrast images for “Harmony vs. others (Neutral and Disharmony)” and “Disharmony vs. others (Neutral and Harmony)” were run through a second-level *t*-test to make statistical maps at the group level using a random effect model. The statistical threshold was set at *p* < 0.005 at the voxel level. We reported the cluster level activations with *p* < 0.05 FWE (family-wise error) correction. And then we conducted an analysis using small volume correction within anatomical mask of the bilateral amygdala made by WFU_PickAtlas (Maldjian et al., 2003, 2004). It allowed investigating whether viewing negative-valenced stimuli induced an activation of amygdala.

## Results

### Preliminary Psychophysical Experiment

**Figure 3:**
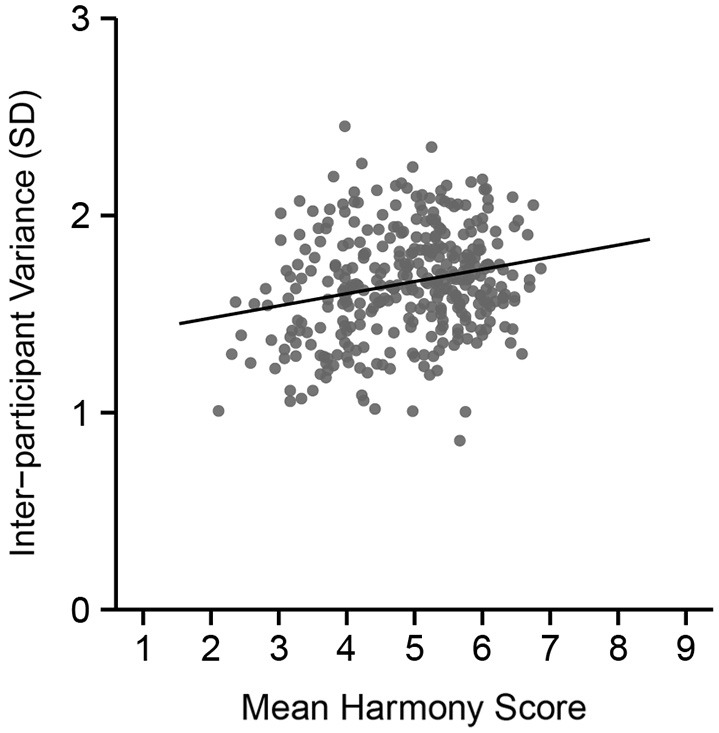
**Inter-participant variance in color harmony score during the preliminary psychophysical experiment across mean harmony scores**. The vertical axis indicates the size of the standard deviation in color harmony scores across the 18 participants.

The mean harmony score (based on a 9-point scale) for each color combination was calculated, and we divided all combinations into three groups. Figure 3 shows inter-participant variance in harmony scores over the 351 combinations. Results from a correlation analysis revealed a positive correlation between inter-participant variance and mean harmony score (*r* = 0.22, *t*_(349)_ = 4.34, *p* < 0.0001). This suggests that disharmonious combinations had a small variance in ratings among participants compared with harmonious combinations.

### fMRI Experiment

**Figure 4:**
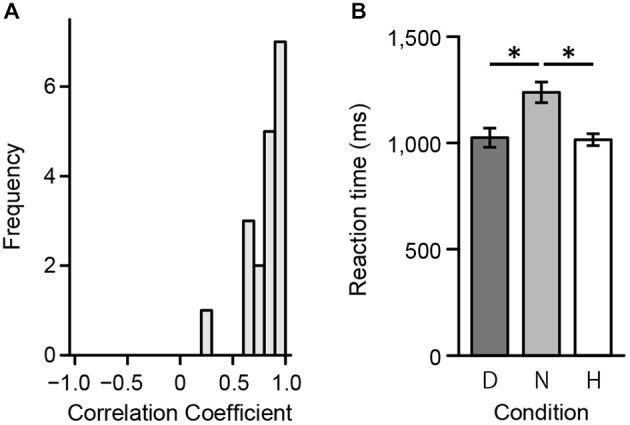
**Behavioral data from the fMRI experiment. (A)** Correlation coefficients between the preliminary psychophysical and fMRI experiment and **(B)** mean reaction times during the fMRI experiment across the three conditions (Harmony, Neutral, and Disharmony). Each error bar indicates standard error of mean (SEM). *p < 0.05.

First, to assess consistency in ratings between the preliminary psychophysical and fMRI experiment, we calculated individual polychoric correlation coefficients. Polychoric correlations were used since ratings during the scan session included a 3-point scale. Figure 4A displays the distribution of individual coefficients. A one-way repeated measures ANOVA was then conducted to evaluate response times in the scanner. There was a significant main effect of reaction time (*F*_(2,34)_ = 22.41, *p* < 0.001). Results from a multiple comparisons test using Shaffer’s modified Bonferroni procedure (with the same procedure as described below for performing multiple comparisons) showed a significant difference between Harmony and Neutral (*t*_(17)_ = 4.95, *p* < 0.001) and between Disharmony and Neutral conditions (*t*_(17)_ = 6.10, *p* < 0.001; Figure 4B). These results indicate that participants took longer when classifying a color combination as neutral.

**Figure 5:**
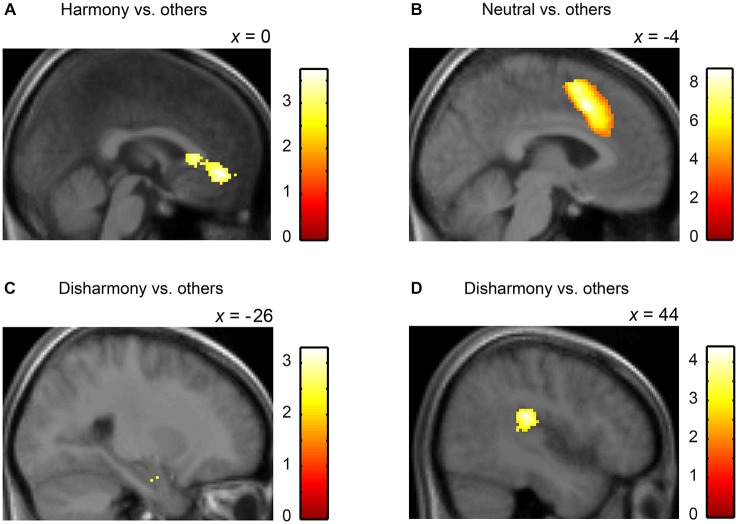
**Statistical parametric maps rendered on a mean T1-weighted image (*N* = 18)**. Harmony vs. others: **(A)** left medial orbitofrontal cortex and anterior cingulate cortex (Talairach coordinates: 0, 39, −4). Neutral vs. others: **(B)** left anterior cingulate cortex (−4, 14, 40). Disharmony vs. others: **(C)** left amygdala (−26, −7, −18) and **(D)** right posterior insula (44, −28, 22).

We converted MNI coordinates obtained in SPM8 to Talairach coordinates and then specified anatomical labels of activation using the Talairach atlas (Talairach and Tournoux, 1988). Table 2 shows activated regions during presentation of Harmony, Neutral, and Disharmony stimuli when compared to other stimuli as a baseline. During the presentation of Harmony stimuli, the bilateral rostral anterior cingulate cortex (rACC) and medial orbitofrontal cortex (mOFC) was significantly active, while the left amygdala and right posterior insula were significantly active during the presentation of Disharmony stimuli. The presentation of Neutral stimuli activated the caudal ACC (cACC; Figure 5). However, correlation analyses (H > N > D; D > N > H) revealed no significant voxels at the prescribed significance level (*p* > 0.005, uncorrected).

**Table 2:** **Activated brain regions for each contrast**.

Activation	L/R	BA	Cluster-level		Voxel-level				Talairach coordinates		
			*p* (FWE)	*k*E	*p* (FWE)	*p* (unc)	*T*	*Z*	*x*	*y*	*z*
**Harmony vs. others (*p > 0.005, uncorrected)***
Anterior cingulate cortex	L/R	24/32	0.042	379	0.954	0.000	3.78	3.59	0	39	−4
Medial orbitofrontal cortex	L	10			1.000	0.001	3.39	3.24	−6	54	−3
**Disharmony vs. others (*p > 0.005, uncorrected)***
Posterior insula	R	–	0.022	438	0.432	0.000	4.39	4.11	44	−28	22
**Disharmony vs. others with small volume correction (*p > 0.001, uncorrected)***
Amygdala	L	–	0.030	5	0.011	0.000	3.81	3.61	−26	−7	−18
**Neutral vs. others (*p > 0.001, uncorrected)***
Anterior cingulate cortex	L/R	32/6	0.000	4310	0.000	0.000	8.38	6.92	−4	14	40
Medial globus pallidus	L	–	0.000	585	0.000	0.000	7.00	6.05	−14	−4	4
Anterior insula	R	–	0.000	403	0.000	0.000	6.69	5.84	32	18	5
Middle frontal gyrus	L	46	0.000	778	0.003	0.000	6.00	5.36	−36	23	26
Inferior parietal lobule	L	40	0.000	443	0.021	0.000	5.44	4.94	−30	−43	37
Middle frontal gyrus	R	46	0.000	732	0.072	0.000	5.05	4.63	30	46	20
Cerebellum	R	–	0.028	179	0.125	0.000	4.87	4.49	26	−59	−22
Middle frontal gyrus	L	46/10	0.000	789	0.204	0.000	4.70	4.35	−32	59	8

*The statistical threshold was set at *p* < 0.05, FWE-corrected at the cluster level. Based on prior knowledge, we applied a small volume correction within an anatomical mask comprising the bilateral amygdala (*p* < 0.001, uncorrected, voxel-level). BA, Brodmann area*.

**Figure 6:**
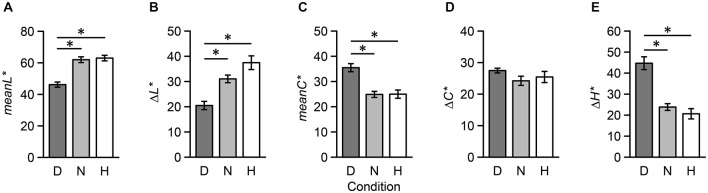
**Five psychophysical indices of color combination stimuli used during the fMRI experiment across the three conditions**. These indices were based on CIELAB metrics: **(A)** mean lightness, **(B)** difference in lightness, **(C)** mean chroma, **(D)** difference in chroma, **(E)** difference in hue. Each error bar indicates SEM. *p < 0.05.

To quantify the perceptual distance of two colors, we introduced five psychophysical indices: difference in lightness (Δ L*), mean lightness (meanL*), difference in chroma (Δ C*), mean chroma (meanC*), and difference in hue (Δ H*) (Note: we avoided using mean hue since we determined it to be an unsuitable psychophysical index; for example, the mean hue of red and green is yellow). Results from one-way repeated measures ANOVAs revealed that main effects of meanL* (*F*_(2,34)_ = 24.44, *p* < 0.001), Δ L* (*F*_(2,34)_ = 14.08, *p* < 0.001), meanC* (*F*_(2,34)_ = 13.02, *p* < 0.001), and (Δ H*) (*F*_(2,34)_ = 23.04, *p* < 0.001) were significant; however, the main effect of Δ C* (*F*_(2,34)_ = 1.10, *p* = 0.344) was not significant. Additionally, multiple comparisons revealed that there were no significant differences between Neutral and Harmony stimulus combinations (*p* > 0.05; Figure 6).

## Discussion

The current study revealed that the left mOFC was activated during the presentation of a harmonious color combination, while the left amygdala and right posterior insula were activated during perception of a disharmonious color combination. Observing harmonious stimulus combinations activating the mOFC is consistent with previous studies showing that this region is activated when individuals are immersed in an esthetically pleasing experience. For example, in a musical context, paralimbic areas, including the medial prefrontal and orbitofrontal cortex, are activated by consonant (harmonious) sounds (Blood et al., 1999). In a visuo-spatial context, esthetically pleasing paintings tend to activate the mOFC, and this activation is associated with increased esthetic preferences (Kawabata and Zeki, 2004). Ishizu and Zeki (2011) demonstrated that the mOFC is activated during experiences of musical and visual beauty. Furthermore, the mOFC is activated when an attractive face is presented (O’Doherty et al., 2003). Therefore, mOFC activation is not restricted to experiences of color harmony but reflective of various esthetic experiences regardless of modality.

Based on results from the “Harmony vs. others” contrast, the cluster including the mOFC was extended to the rACC. The ACC is part of the limbic lobe and is functionally divided into rostral and caudal portions (Bush et al., 2000). The ACC is part of a circuit involved in attention serving to regulate both emotional (rACC) and cognitive (cACC) processing. The rACC is also suggested to be responsive toward positive, esthetic appraisals (Brown et al., 2011). Furthermore, Vartanian and Goel (2004) demonstrated that activation within the rACC correlates with preference ratings. The present findings regarding cACC activation might have reflected cognitive conflict during the Neutral condition. Longer reaction times compared to the Harmony and Disharmony conditions indicate that initial responses help determine whether the combination is harmonious or disharmonious. Thus, a stimulus that was not harmonious or disharmonious might be judged as neutral.

We also observed that disharmonious stimulus combinations activated the left amygdala and right posterior insula. The amygdala plays an important role in evaluating the biological significance of affective visual stimuli. For instance, the amygdala is significantly activated when emotionally negative stimuli are presented (Davis and Whalen, 2001), and activation in the amygdala might involve automatic processing of affective visual stimuli (Pessoa and Adolphs, 2010). The insula is also involved in the processing of negative emotion, particularly fear, sadness, and disgust (Phillips et al., 1998). For example, fear-related pictures increase activation in the right posterior insula and secondary somatosensory cortex (Straube and Miltner, 2011). Taken together, activation within the amygdala and insula in response to disharmonious combinations might have important biological implications.

Results from the preliminary psychophysical experiment revealed that individual differences in color harmony scores were less variable during the presentation of disharmonious stimuli than neutral and harmonious stimuli. Moreover, perceptual characteristics of disharmonious stimuli were different from those of neutral and harmonious stimuli during the fMRI experiment. Consequently, these results suggest that a disharmonious color combination is mostly determined at a lower perceptual level and is not affected by higher cognitive factors contributing to individual differences (as opposed to a neutral or harmonious combination). In other words, disharmonious combinations can be determined by perceptual characteristics, including darkness, saturation, and a complementary combination. However, since there was no clear difference in perceptual characteristics between neutral and harmonious combinations, larger individual differences might result when discriminating as to whether a color combination is harmonious or neutral.

Previous studies have treated the scoring of color harmony as a single, bipolar scale; however, results of the present psychophysical and fMRI experiments suggest that color harmony contains two processes. First, color disharmony depends on the stimulus itself and automatic neural processing that is mediated by the amygdala and insula. In contrast, color harmony is hard to determine based on color characteristics alone, and is supported by the processing of esthetic value within the mOFC. This asymmetry has been reported in other research domains. For instance, Baumeister et al. (2001) reported that negative events have a greater influence on human cognition than positive events. Therefore, this asymmetry could be highly adaptive for avoiding painful events relevant for survival. Thus, in addition to esthetic preference, disharmonious color combinations might serve a biological purpose (e.g., warnings based on certain color combinations; Stevens and Ruxton, 2012).

Color combination is a simple stimulus component composed of low-level features. Previous neuroesthetic studies have examined low-level stimuli, including symmetric geometrical shapes (Jacobsen et al., 2006) or moving dots (Zeki and Stutters, 2012). These studies revealed that preferred stimuli activate clusters that include the mOFC and frontal pole. Additionally, those studies observed that preferred motion stimuli activated V5, which is extensively responsible for motion processing in the visual cortex. Activation within the visual cortex was explained by the physical characteristics of motion stimuli or the influence of top-down attention from the prefrontal cortex. The present results showed that V4 was not activated within any contrasts. This could be the result of no differences in chromatic contrast across the three conditions (Figure 6D) if physical characteristics cause activation within V4. Furthermore, the amygdala (Vuilleumier, 2005) and the medial part of superior frontal cortex (Hopfinger et al., 2000; Kastner and Ungerleider, 2000) were also the source of top-down attention during the Disharmony and Neutral conditions. Additionally, this inconsistency could have emerged due to differences between preference and harmony.

The present study has a couple of limitations to note. The first relates to statistical power for the fMRI data analysis. Using a significance cut-off of *p* < 0.005 in the “Harmony vs. others” contrast was somewhat liberal compared with other fMRI studies. The second limitation is that the experimental design used cannot exclude the effect of preferences since we did not collect data regarding color preference judgments from the same participants. Schloss and Palmer (2011) reported that a preferred color combination tends to have a significant lightness contrast. However, we were unable to observe a difference between Neutral and Harmony condition based on a lightness contrast. Furthermore, the task instructions focused on harmony, not preferences. However, to determine whether the issue of preference effects is notable, future studies should directly compare across both harmony and preference judgments.

A distinction between beauty and preference has not been fully clarified within the field of neuroesthetics. Individual differences and expertise are important variables that could help provide a clearer understanding between differences in harmony (even beauty) and preference judgments in the brain.

## Conflict of Interest Statement

The authors declare that the research was conducted in the absence of any commercial or financial relationships that could be construed as a potential conflict of interest.
